# Does Religious Involvement Mitigate the Effects of Major Discrimination on the Mental Health of African Americans? Findings from the Nashville Stress and Health Study.

**DOI:** 10.3390/rel8090195

**Published:** 2017-09-17

**Authors:** Christopher G. Ellison, Reed T. DeAngelis, Metin Güven

**Affiliations:** College of Liberal and Fine Arts, The University of Texas at San Antonio, San Antonio, TX 78249, USA.

**Keywords:** major discrimination, African Americans, mental health, depression, life satisfaction, coping, stress process, religious involvement

## Abstract

Several decades of scholarly research have revealed the significant toll of discrimination experiences on the well-being of African Americans. Given these findings, investigators have become increasingly interested in uncovering any potential resources made available to African Americans for mitigating the psychosocial strains of discrimination. The current study contributes to this literature by testing whether various indicators of religious involvement – e.g. church attendance, prayer, and religious social support – buffer the noxious effects of major discrimination experiences on the mental health outcomes (i.e. depression and life satisfaction) of African Americans. We analyze data from the African American subsample (n=627) of Vanderbilt University’s Nashville Stress and Health Study, a cross-sectional probability sample of adults living in Davidson County, Tennessee between the years 2011 and 2014. Results from multivariate regression models indicated: (1) experiences of major discrimination were positively associated with depression and negatively associated with life satisfaction, net of religious and sociodemographic controls; and (2) religious social support offset and buffered the adverse effects of major discrimination on both mental health outcomes, particularly for those respondents who reported seeking support the most often. We discuss the implications and limitations of our study, as well as avenues for future research.

## 1. Introduction

Despite the advances of the civil rights movement, discrimination remains an ugly reality for millions of African Americans. Discrimination in American society can take a plethora of forms, ranging from unequal treatment in housing, employment, and other public and institutional arenas, to insults and overt hostility, to more subtle slights and daily micro-aggressions (Bertrand and Mullainathan 2004; Fix and Turner 1999; Harris et al. 2005; Roscigno 2007; Ross and Yinger 2002; Williams, Nesiba, and McConnell 2005). The continuing reality of discrimination has been documented through various means, including (a) surveys of African Americans’ experiences and perceptions, (b) audits conducted by governmental agencies and civil rights groups, (c) experimental studies, and (d) analyses of broad statistical populations that document unequal effects in institutional practices (for a review, see Pager and Shepherd 2008). Over the past two decades, investigators have come to recognize the significant toll of discrimination on the physical and emotional well-being of African Americans (Williams and Mohammed 2009; Williams and Williams-Morris 2000). In addition, experiences of discrimination have been linked with depression, anxiety, distress, anger, and other negative mental health outcomes (Berger and Saranyai 2015; English, Lambert, and Ialongo 2014; Kwate and Goodman 2015).

Given such findings, researchers have shown considerable interest in social and psychological factors that may mitigate the harmful effects of discrimination on well-being within the African American population. Theoretical and sociohistorical works have suggested that religion may aid African Americans in dealing with the emotional consequences of discrimination (Cooper-Lewter and Mitchell 1986; Gilkes 1980; Griffith, English, and Mayfield 1980). Nevertheless, although there is considerable evidence that religious involvement is related to salutary mental health outcomes for African Americans (for a review, see Ellison et al. 2010), relatively few empirical studies have examined the role of religion in buffering the effects of discriminatory experiences, and the findings to date have been somewhat discrepant (Bierman 2006; Ellison, Musick, and Henderson 2008; Head and Thompson 2017; Hope et al. 2017). In light of these patterns, further research is needed regarding *which* specific aspects of religious involvement moderate the deleterious impact of *which* specific types of discrimination.

Our study contributes to this literature in several ways. First, we focus squarely on the effects of major discrimination – particularly unequal treatment encountered in public settings and institutional contexts (e.g., education, labor markets, and housing) – on two important mental health outcomes: depressive symptoms and life satisfaction. We also explore the role of multiple facets of religion, including religious attendance, prayer, and congregational support. Relevant hypotheses are assessed using data from the African American subsample (n=627) of the Nashville Stress and Health Study (hereafter NSAHS), a probability sample drawn in Davidson County, TN between 2011 and 2014. After presenting the results, we discuss the implications of the findings, study limitations, and promising directions for future inquiry.

## 2. Theoretical and Empirical Background

### 2.1. Discrimination and Mental Health

A growing body of research has documented robust associations between experiences of discrimination and African Americans’ mental health (English et al. 2014; Kessler et al. 1999; Kwate and Goodman 2015). These patterns may be caused by several factors. Unequal treatment in institutional contexts such as education, labor markets, consumer markets, credit, health care, and others can involve material deprivation and the loss of valuable resources (Bertrand and Mullainathan 2004; Fix and Turner 1999; Harris et al. 2005; Roscigno 2007; Ross and Yinger 2002; Williams et al. 2005). This can threaten the security and quality of life, as well as the life chances of loved ones. Unequal treatment – usually, but not exclusively, at the hands of the majority population – may also be hurtful because it calls into question one’s sense of self, and challenges one’s worthiness and status as a full citizen. This can result in feelings of helplessness, alienation, frustration, and other negative emotions. Experiences of discrimination can also serve as painful reminders of injustices suffered by others, including members of one’s family and community. Thus, many African Americans feel the need to remain vigilant and alert for negativity and unequal treatment, and to devote valuable energy to assessing the intentions of others, and – in the event that discrimination is detected – to consider response options (Himmelstein et al. 2015). For all of these reasons, experiences of discrimination may erode African Americans’ emotional well-being, promoting depression and distress, and calling into question perceived progress toward major life goals.

### 2.2. African American Religion as a Stress Buffer

Numerous previous discussions, and a limited body of empirical research, have suggested that religion may play a role in shielding African Americans from the deleterious psychological consequences of discrimination and other stressful circumstances (Bierman 2006; Ellison 1993; Ellison, Musick, and Henderson 2008; Henderson 2016). This makes sense for a number of reasons. Religion has played a prominent role in the collective and individual lives of African Americans throughout U.S. history (Billingsley 1999; Lincoln and Mamiya 1990; Taylor, Chatters, and Levin 2004). The Black Church has been central to the African American experience because it has traditionally been one of the few institutions that has been ubiquitous and organized solely by and for African Americans. Religious groups have served as fertile ground for the development of African American community and political leadership, economic development efforts, and social insurance initiatives, among many other functions (Billingsley 1999; Lincoln and Mamiya 1990). Over the years, studies have repeatedly shown that African Americans (as well as Caribbean-born Blacks) are more religious, by virtually any conventional indicator, than White Americans from otherwise comparable backgrounds (Chatters et al. 2009; Taylor, Chatters, and Levin 2004). This is the case with regard to both organizational involvement (e.g., denominational and congregational membership, religious attendance and church participation) and non-organizational practices (e.g., prayer, scriptural study, religious media consumption). In addition, most African Americans agree that the Black Church has played an important and highly beneficial role in sustaining community and individual well-being in the face of inequality, racial exclusion, and social marginality (Krause 2004a; Taylor, Thornton, and Chatters 1987).

How and why might religious involvement mitigate the effects of discrimination on African Americans’ emotional well-being? The past thirty years have seen a remarkable growth in the research literature on the links between religion and health, particularly mental health (Ellison and Henderson 2011; Koenig, Carson, and King 2012). A number of these studies have documented associations between religious factors and various mental health outcomes among African Americans, including: life satisfaction (Ellison and Gay 1990; Levin, Chatters, and Taylor 1995); psychological distress (Oates and Goode 2013; Tabak and Mickelson 2009); depressive symptoms and major depressive disorder (Chatters et al. 2011; Ellison and Flannelly 2009; Taylor, Chatters, and Abelson 2012), suicidal ideation and suicidal behavior (Chatters et al. 2015; Taylor, Chatters, and Joe 2011), and others (Chatters et al. 2008). It is well-established that religion is a complex, multidimensional phenomenon, and researchers routinely distinguish, at a minimum, between organizational practices (e.g., attendance at services, participation in congregational activities) and non-organizational behaviors (e.g., prayer, scriptural reading). More recently, scholars have also identified a number of facets of religious involvement that are uniquely linked with health outcomes (Idler et al. 2003; Krause 2008a; Pargament, Koenig, and Perez 2000). For example, investigators increasingly recognize the importance of church-based social support for health and well-being, particularly for African Americans (Nguyen, Chatters, and Taylor 2016). Consideration of these dimensions of religion is consistent with the broad reflection on the nature of African American religious life offered by Mattis and Jagers (2001): “African American religiosity and worship traditions emphasize a profound sense of intimacy with the divine, and a horizontal extension of that intimacy into the human community” (523).

#### 2.2.1. Organizational Religiosity

Organizational religiosity, and particularly religious attendance, may be linked with mental health for several reasons (Ellison 1991; Lim and Putnam 2010). Briefly, worship services bring together individuals who share common religious convictions – and frequently, social values and status characteristics – in activities and rituals to which they ascribe sacred significance. These practices may further strengthen religious meaning systems and what Berger (1967) famously termed “plausibility structures.” Regular participation in organizational religious activities often builds solidarity and trust among fellow believers, and offers a rich context for the development of friendship networks, and, for many persons, a significant outlet for social participation (Ellison and George 1994). Informal interactions with coreligionists may reinforce religiously informed understandings of social roles (e.g., in families and in civic life), and may affirm shared understandings of personal and social problems (Ellison 1993; Krause 2008).

Church involvement has been especially important among many African American communities, giving rise to strong social norms of religious affiliation and involvement, and these activities have often served as gateways to respectability, status, and community leadership (Ellison and Sherkat 1995). Church members often play prominent roles in the social networks of African Americans (Nguyen, Chatters, and Taylor 2016; Taylor, Chatters, and Levin 2004). In addition, African Americans’ religious attendance may facilitate emotional well-being due to the distinctive modes of worship that prevail in many (though certainly not all) predominantly Black congregations (Edwards 2009). For example, religious services often feature dynamic preaching, and worship styles that involve rich music and energetic singing and dancing, shouting, and other forms of participation that are geared toward the release of negative emotions (e.g., sorrow, frustration, guilt) and the cultivation of positive feelings (Gilkes 1980; Griffith, English, and Mayfield 1980). Taken together, these lines of argument suggest that African Americans who attend services regularly may enjoy greater life satisfaction, and may experience fewer harmful emotional consequences from discrimination, than their less involved counterparts.

#### 2.2.2. Non-Organizational Religiosity

Religious practices that take place outside of institutional settings, particularly prayer, may be associated with mental health as well. Briefly, individuals construct personal relationships with a divine other (i.e., God, Jesus) in much the same way they build concrete social bonds (Pollner 1989; Sharp 2010). To be sure, prayer can take many different forms, including ritual prayer (e.g., praying the Rosary), contemplative or meditative prayer, and others (e.g., Krause and Chatters 2005). However, for many persons, prayer is experienced as a conversation – and often as an ongoing dialogue – with a divine other (Pollner 1989; Sharp 2010). Individuals derive an understanding of the nature of the divine through religious socialization and training, scriptural stories and other passages, sermons, and narratives and testimonials from contemporary figures. From these sources, believers can gain a sense of how God relates to His creation, and what is expected of them in terms of devotion, faith, and behavior (Pollner 1989). In this way, prayer is experienced as an interaction with a divine other, by which an intimate, individual relationship is sustained and nurtured. Indeed, prayer can be an important resource for coping with stressful events and conditions. In this context, many individuals cultivate what they perceive to be collaborative partnerships with a divine other. This ability to interact with an imagined deity who is both omnipotent and benevolent can aid and augment one’s own efforts to manage negative emotions and solve personal difficulties (Pargament 1997; Pargament, Koenig, and Perez 2000). Although the evidence is not unequivocal, a number of studies have linked prayer with psychological well-being, especially when that prayer is thought to involve a benevolent, forgiving deity and a warm and secure relationship, without demands or expectations for specific outcomes (Krause 2004b; Ellison et al. 2014).

Although these insights are germane to mental health within the general population, they may be especially relevant for African Americans, who tend to engage in prayer and other devotional practices more often than Whites (Chatters et al. 2009; Taylor, Chatters, and Levin 2004). A rich African American theological tradition has tended to view God as omnipotent and omniscient, and has tended to emphasize God’s love, compassion, and mercy (Cooper-Lewter and Mitchell 1986; Washington 1994). For many African American Christians, God is good and gracious. Mainstream African American theology has often centered on themes of triumph over personal misfortune and spiritual redemption, along with individual and collective liberation from oppression (Roberts 2005). The faith traditions of many African Americans have stressed a close and deeply personal relationship with God (Washington 1994). To many observers, this is a highly practical theology that is geared toward helping African Americans cope with generations of racism and marginality (Mattis and Jagers 2001; Roberts 2005). Research shows that many, perhaps most, African Americans turn to religion and spirituality to manage or regulate negative emotions that arise due to stressful events and conditions, and that religious coping practices are experienced as successful (Ellison, Musick, and Henderson 2008; Ellison and Taylor 1996). For all of these reasons, it is reasonable to expect that African Americans who pray more frequently will report better mental health, and will sustain less emotional damage from experiences of discrimination than others.

#### 2.2.3. Church-Based Social Support

A wealth of research over the years has established that social support has robust positive effects on well-being, and can buffer the deleterious effects of stressful events and conditions on mental health outcomes (Krause 2008a). A growing body of theory and evidence underscores the importance of religious congregations as conduits of various types of social support (Krause 2002, 2008a; Nguyen, Chatters, and Taylor 2016). To be sure, congregations may offer several types of formal support, via ministries targeted at specific segments of the membership, and through programs to assist those in need, ranging from aid to the poor, ill and disabled, families in crisis, and others (Billingsley 1999; Tsitsos 2003). In addition, clergy members provide help through pastoral counseling, which often addresses mental health issues or family matters (Neighbors et al. 1998; Taylor et al. 2000), but potentially addresses a much broader array of issues affecting the lives of church members.

However, a great deal of church-based social support occurs through informal exchanges among members themselves, and between members and clergy (Krause 2003). Briefly, religious groups are network-driven institutions: individuals and families are often recruited into congregations by pre-existing social ties, and once they join, congregational settings allow them to form and sustain lasting social relationships (Ellison and George 1994; Lim and Putnam 2010). Religious cultures typically encourage kindness and helping behavior toward fellow members (and others), as well as norms of reciprocity, and individuals and families often belong to congregations for periods of years. Over time, members can cultivate support convoys, or accumulations of social relationships with the potential to deliver assistance in times of difficulty (Ellison and George 1994). Indeed, church members may exchange multiple types of support, ranging from instrumental or tangible aid (e.g., goods, services, and information) to socioemotional assistance (e.g., companionship and morale support, love and caring) (Krause 2002, 2008a). Although such social support may be obtained from other sources (e.g., relatives, friends, neighbors, and coworkers), there is at least some evidence that assistance from religious sources may confer greater mental and physical health benefits (e.g., Krause 2006).

Church-based social support may be particularly important for African Americans. On average, they tend to exchange instrumental and socioemotional aid informally with fellow church members more often than Whites from comparable backgrounds (Krause 2002, 2008). Several studies report that such congregational support, particularly socioemotional assistance, is linked with health and well-being among African Americans (Chatters et al. 2011, 2015; Ellison, Musick, and Henderson 2008; Head and Thompson 2017; Hope et al. 2017), perhaps more so than among Whites (Krause 2003, 2008a). Among African Americans, church-based support often augments and complements – rather than replicates – the support that is available from family members and other non-kin ties (Nguyen, Chatters, and Taylor 2016). Indeed, some studies have reported that support from church members helps to explain much of the salutary association between African Americans’ religious participation and psychological distress (Holt et al. 2014; Jang and Johnson 2004). Moreover, in addition to the prominent role of church members, clergy members are particularly important sources of varied types of support among African Americans (Krause 2003; Neighbors, Musick, and Williams 1998; Taylor et al. 2000). Pastors may be especially significant members of support networks because they are common, respected and trusted for their spiritual insights and worldly knowledge, and accessible to persons with limited means. Given the foregoing, it is reasonable to suspect that African Americans who seek and receive support from church members more frequently will be less vulnerable to the damaging emotional effects of discrimination.

## 3. Conceptual Models

**Figure 1.**
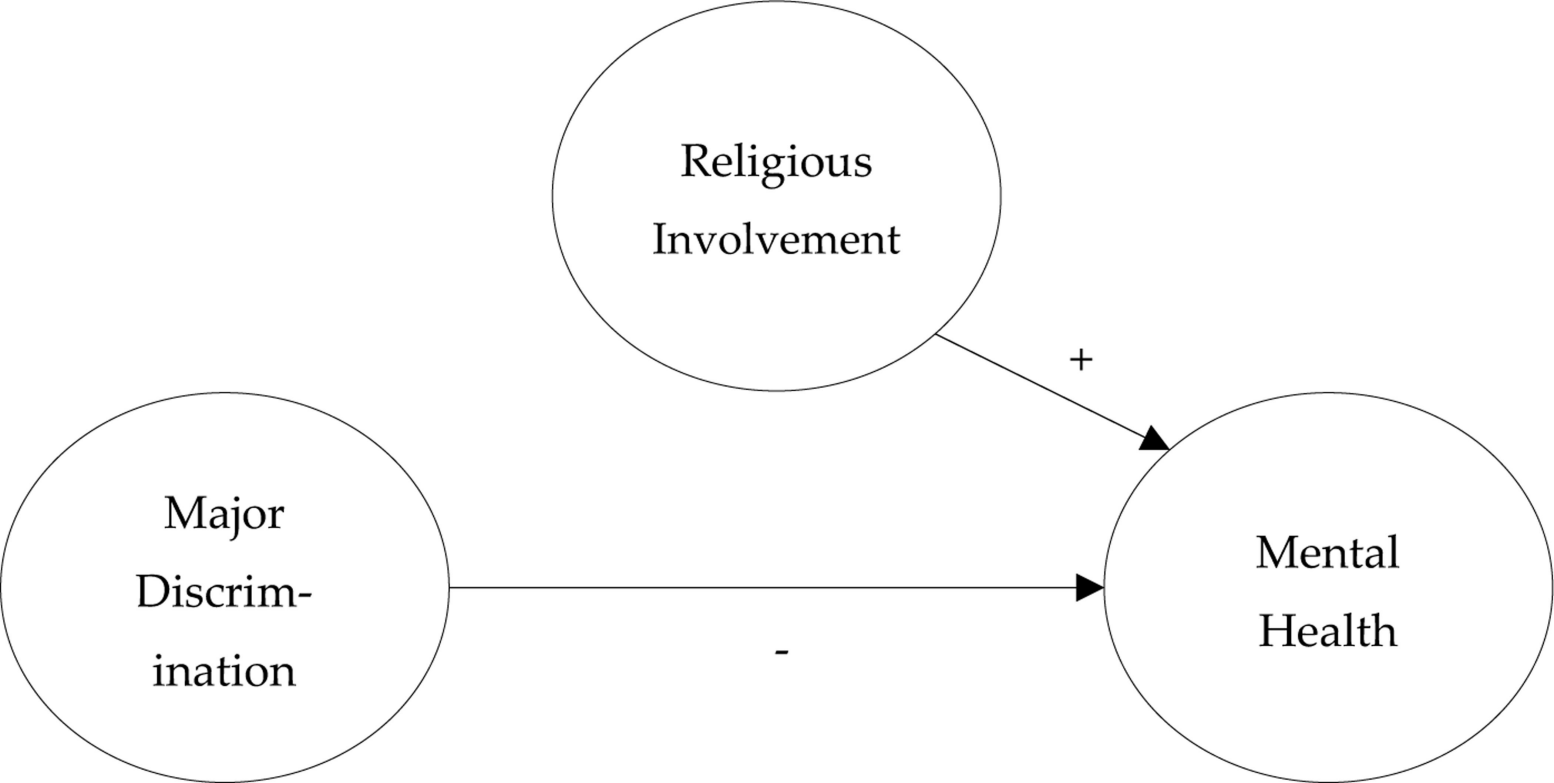
Stress-Offsetting Conceptual Model.

**Figure 2.**
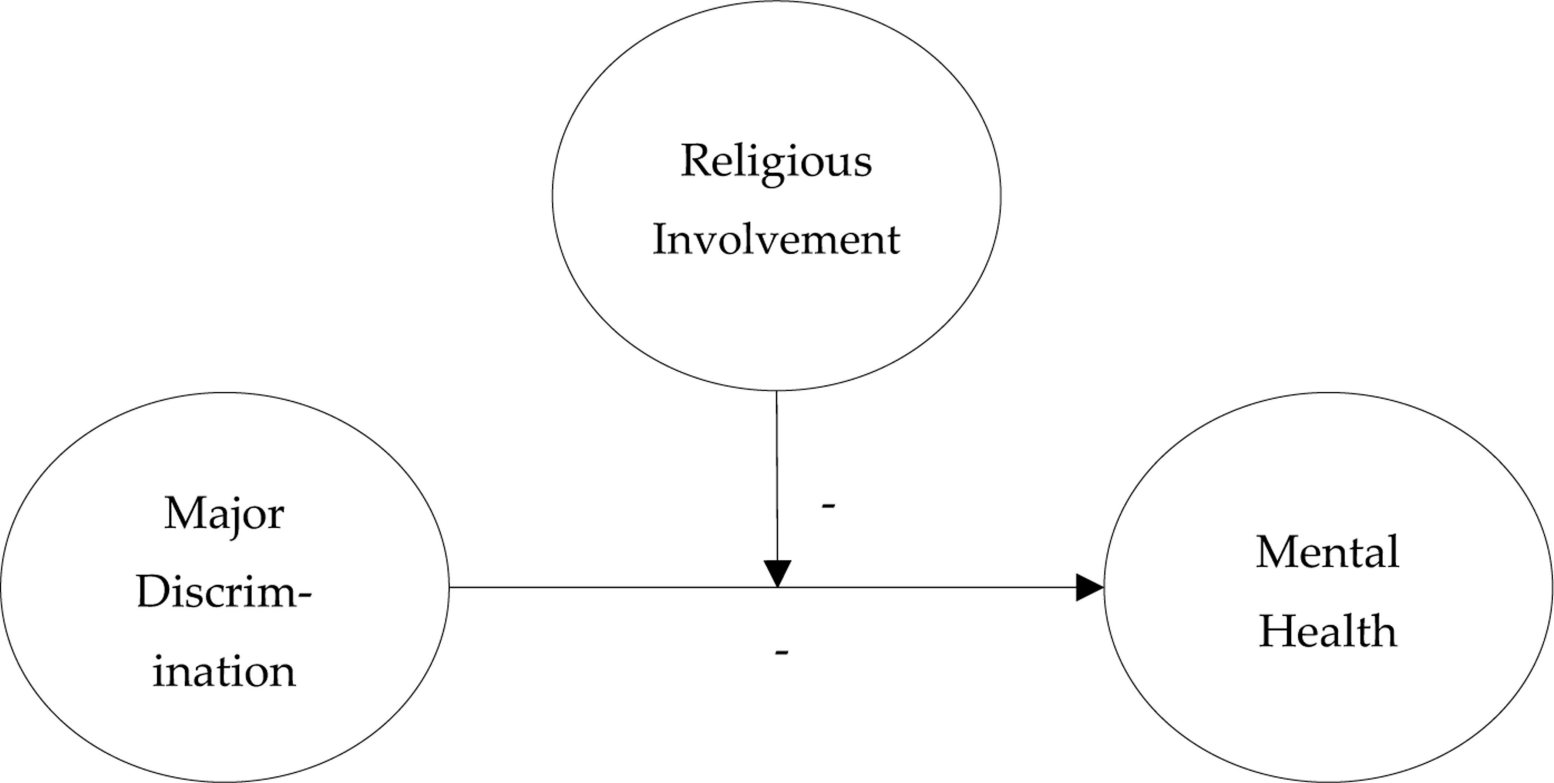
Stress-Buffering Conceptual Model.

Our study hypotheses can be summarized within a general stress-process conceptual scheme (see Ellison and Henderson 2011). Our first hypothesis, pictured below in Figure 1, suggests that religious involvement will contribute its own salutary mental health effects, in turn offsetting the adverse effects of major discrimination on mental health (i.e. stress-offsetting hypothesis). Our second hypothesis, pictured below in Figure 2, suggests that religious involvement will *interact* with discrimination experiences to buffer the negative associations between major discrimination and mental health (i.e. stress-buffering hypothesis). Put another way, the adverse mental health effects of major discrimination experiences should *diminish* as a function of increased religious involvement. Moreover, our two hypotheses, though distinct, are not mutually exclusive. Religious involvement could both offset *and* buffer the psychosocial strains of major discrimination experiences.

## 4. Methods

### 4.1. Data

To explore the possible role of these multiple dimensions of religious involvement in buffering the deleterious effects of discrimination experiences on African Americans’ mental health, we analyze data from Vanderbilt University’s Nashville Stress and Health Study (NSAHS; 2011–2014). The NSAHS is a probability sample of non-Hispanic black and white women and men aged 22 to 69 living in Davidson County, Tennessee (http://www.vanderbilt.edu/stressandhealthstudy). The primary research objective of the NSAHS was to investigate health differentials rooted in racial and socioeconomic disparities. The NSAHS survey included items measuring family background, experiences of discrimination and other psychosocial stressors, physical and mental health outcomes, and religious engagement, to name only a few. The NSAHS surveyed 1,252 adults living in a random sample of 199 block groups stratified by the percentage of African Americans assumed to live therein according to 2010 Census data. The sampling frame consisted of 2,400 randomly sampled households and 2,065 eventually were contacted to participate in the study. Nearly 61% of the 2,065 contacted households participated in the study. The interviews were computer-assisted and typically lasted three hours. Interviews were conducted either in the respondent’s home or on Vanderbilt University campus. Trained interviewers conducted the interviews and were matched to respondents based on race. Respondents were offered $50 to participate in the survey interview. Our analyses included only the African American sub-sample of NSAHS respondents (*n* = 627).

### 4.2. Measures

#### 4.2.1. Past-Month Depression

We used the 20-item Center for Epidemiological Studies (CESD) index to gauge respondents’ severity of depressive symptoms. Items asked respondents how often in the past month they were bothered by things that usually did not bother them, could not “shake off the blues,” had trouble keeping their mind on what they were doing, and did not feel like eating, among other symptoms (see Radloff 1977). Response categories ranged from 1 = “not at all” to 4 = “almost all the time.” We averaged the items to create a mean index of past-month depression (α = .92).

#### 4.2.2. Life Satisfaction

We used Diener’s Satisfaction with Life Scale to measure respondents’ life satisfaction (Diener et al. 1985). Respondents answered how much they agreed (4 = “a lot,” 1 = “not at all”) with the following five statements: (1) “In most ways my life is close to my ideal,” (2) “The conditions of my life are excellent,” (3) “I am satisfied with my life,” (4) “So far I have gotten the important things I want in life,” and (5) “If I could live my life over, I would change almost nothing.” We averaged the items to create a mean index of life satisfaction (α = .79).

#### 4.2.3. Major Discrimination

Our discrimination measure was a seven-item checklist inventory of major experiences of unfair treatment (see Kessler et al. 1999). Respondents answered whether they have ever experienced any of the following: (1) been unfairly fired or denied a promotion, (2) not been hired for a job for unfair reasons, (3) been unfairly treated by the police (e.g. stopped, searched, questioned, physically threatened or abused), (4) been unfairly discouraged by a teacher or advisor from continuing education, (5) been unfairly discouraged by a teacher or advisor from pursuing a job/career, (6) for unfair reasons, had a landlord or realtor refuse to sell or rent them a house/apartment, and (7) for unfair reasons, had neighbors make life difficult for them. Response categories were coded such that 1 = “yes” and 0 = “no.” We added yes/no responses to create a checklist inventory of major discrimination experiences.

#### 4.2.4. Religious Involvement

We gauged organizational religious involvement with a single item measuring frequency of attendance at religious services. Respondents were asked, “Which of the following best describes how often you attend services at a church/temple/ synagogue/mosque?” Response categories were coded such that 0 = “never,” 1 = “a few times a year,” 2 = “monthly, and 3 = “weekly or more.” We assessed non-organizational religious involvement with a single item measuring frequency of prayer. Respondents were asked “About how often do you pray?” Response categories ranged from 1 = “never” to 6 = “several times a day.” Finally, we measured religious social support with a single item that asked respondents, “How often do people in your church (place of worship) help you out?” Response categories ranged from 1 = “never” to 4 = “very often.”

Contemporary trends in the religion-health literature suggest that the stress-buffering effects of religion may result from a minimum threshold of engagement, rather than from incremental changes in religious involvement (see Schieman, Bierman, & Ellison 2013). In other words, there is reason to suspect that religious involvement will buffer against the effects of discrimination *only* for those who most frequently attend worship services, pray, and seek religious social support. We test this additional moderation threshold hypothesis with dummy codes for “weekly or more” church attendance, “daily or more” prayer, and seeking religious social support “very often.” For each measure, the reference category is everyone who did not report the maximum engagement value. We provide a separate table of analyses to test this hypothesis (see Table 4 below).

#### 4.2.5. Socio-demographics

Models controlled for age (in years), gender (female = 1, male = 0), education (in years), marital status (1 = married, 0 = not married), employment status (1 = employed, 0 = unemployed), and household income (ordinal, 0 = under $5,000 or less … 15 = $135,000 and above).

### 4.3. Analytic Strategy

We used Stata 13 for all statistical analyses. We estimated both depression and life satisfaction using Ordinary Least Squares (OLS) regression techniques. All models adjusted standard errors for cluster sampling by block group (Stata’s “cluster” command). To test our stress-buffering hypotheses, we created interaction terms between major discrimination and our religious measures of interest. We mean-centered variables before creating interaction terms to help reduce multicollinearity between interaction terms and lower-order coefficients (Aiken, West, & Reno 1991). We also visually depicted statistically significant interactions as linear prediction graphs. These figures display the predicted value of the dependent variable (y-axis) as a function of experiences of discrimination (x-axis) and religious involvement. Finally, the following variables had missing values: depression (n=9), life satisfaction (n=6), frequency of church attendance (n=1), religious social support (n=1), frequency of prayer (n=2), and household income (n=31). For all analyses, we replaced these missing values with five iterations of multiple imputation by chained equation (White, Royston, & Wood 2011).

## 5. Results

Table 1 reports descriptive statistics of study variables among the African American subpopulation of the NSAHS (*n* = 627). Tables 2 and 3 report unstandardized OLS regression coefficients estimating past-month depressive symptoms and life satisfaction, respectively. In both tables, Model 1 reports direct associations between independent and dependent variables. Models 2 through 4 report interaction terms between discrimination experiences, church attendance, prayer, and religious social support, respectively. Table 4 shows results of our moderation threshold test.

First, major discrimination associated positively with past-month depression (*p* < .01) and, to a lesser extent, negatively with life satisfaction (*p* < .10), even after controlling for religious involvement and socio-demographics. In other words, regardless of age, gender, socioeconomic attainment, and religious involvement, respondents who experienced notable episodes of unfair treatment at some point in their life still harbored feelings of distress and dissatisfaction at the time of the interview. These findings are consistent with aforementioned literature and underlie just how destructive experiences of discrimination can be for African Americans.

Second, religious social support was the only religious measure that significantly mitigated and/or offset the negative associations between discrimination and mental health. Model 4 of Table 2 shows that religious social support buffered the positive association between major discrimination experiences and depression (*p* < .05). Likewise, Table 3 demonstrates that religious social support was positively associated with life satisfaction (*p* < .05), and therefore offset the negative association between major discrimination and life satisfaction. Moreover, Models 3 and 6 of Table 4 confirm that religious social support actually buffered the associations between major discrimination and *both* dimensions of mental health (*p* < .05), but only for respondents who reported receiving support “very often.” The same was true for the direct associations between religious social support and mental health. In Table 4, we see that respondents who received religious social support “very often” tended to report significantly less depression (*p* < .05) and greater life satisfaction (*p* < .001) compared with respondents who received support less than very often or not at all.

**Figure 3.**
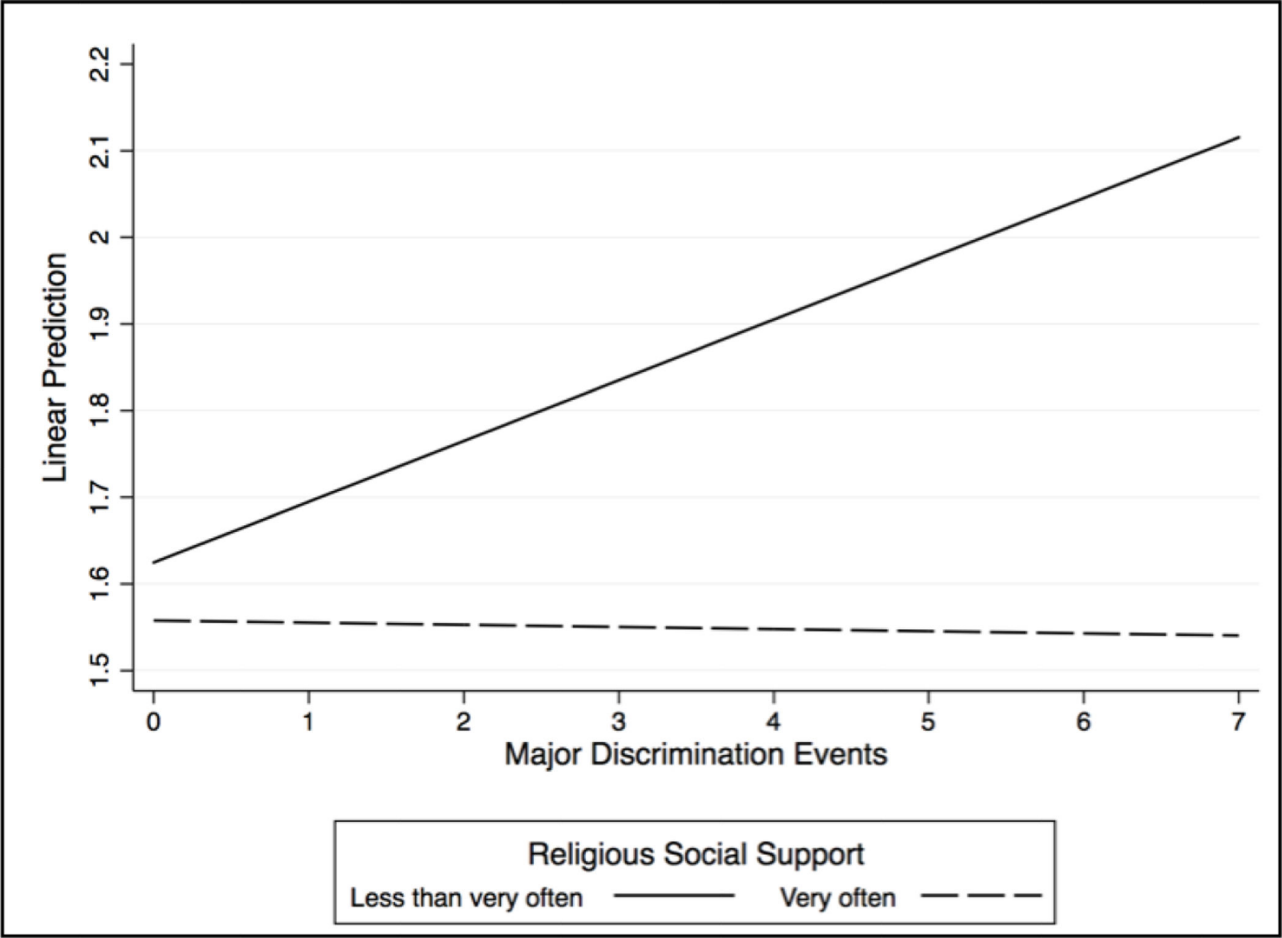
Major Discrimination X Religious Social Support on Depression.

**Figure 4.**
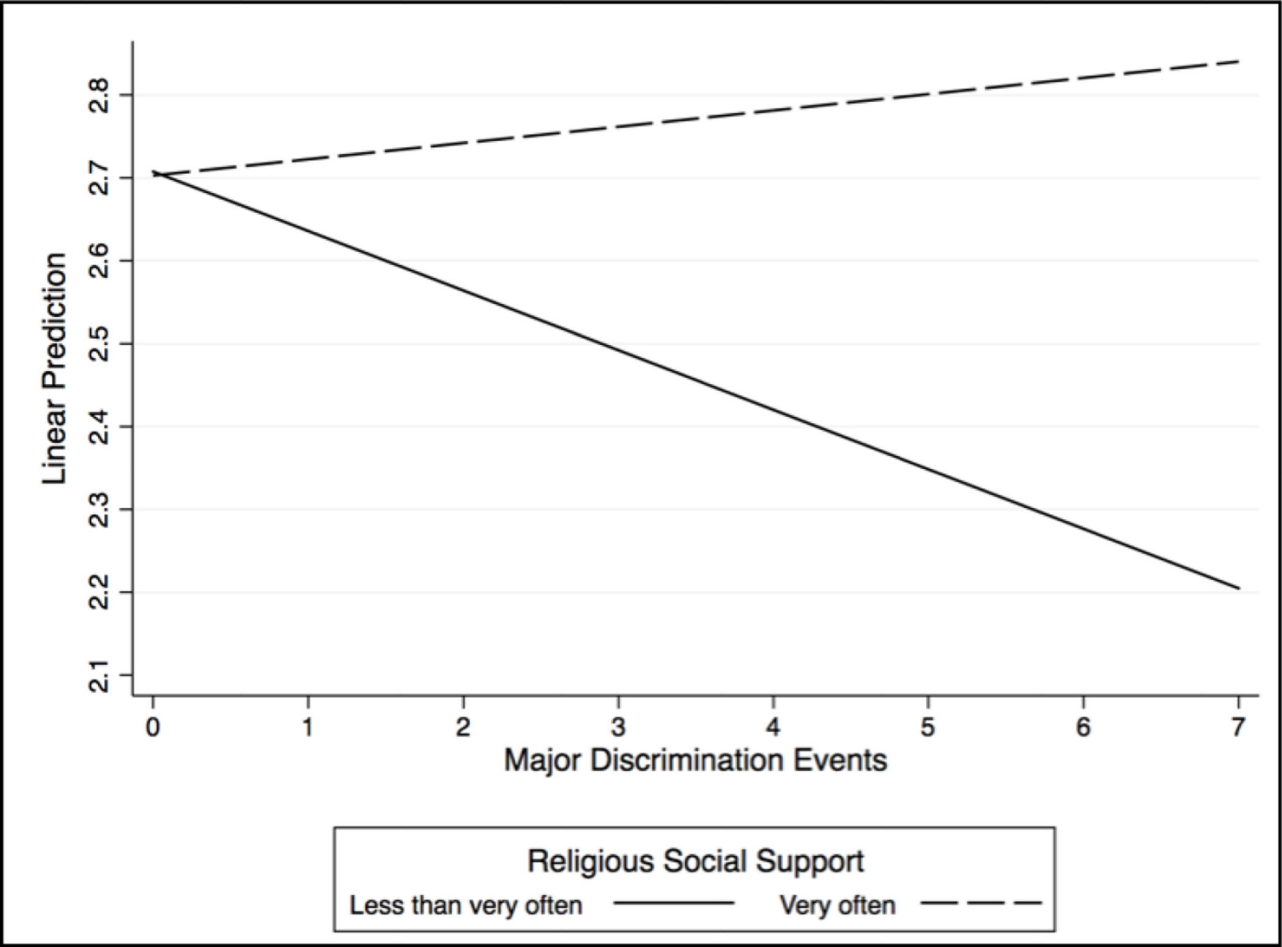
Major Discrimination X Religious Social Support on Life Satisfaction.

Figures 3 and 4 provide a clearer interpretation of our observed moderating patterns. Increased reports of major discrimination events were associated with significant increases in depressive symptoms and decreases in life satisfaction among respondents who said they did *not* receive religious social support “very often.” However, major discrimination events had little to no impact on the mental health outcomes of respondents who reported receiving religious social support “very often.” We discuss these findings in more detail below.

## 6. Discussion and Conclusion

A wealth of recent scholarship has documented the harmful effects of perceived discrimination on the individual health and well-being of African Americans. This has occasioned the search for social and psychological factors that mitigate these deleterious effects, and recent empirical research has focused on the potential role of religion. Our study has contributed to this burgeoning literature by: (1) developing a series of theoretical arguments linking several specific facets of religious involvement – organizational and non-organizational practices and church-based social support – to mental health outcomes among African Americans, with particular attention to potential stress-buffering properties; (2) testing relevant hypotheses using data from a representative sample of African Americans drawn from Davidson County, TN; and (3) exploring both incremental and threshold main and stress-buffering effects of these religious dimensions vis-a-vis two mental health outcomes, depressive symptoms and life satisfaction (e.g. Schieman et al. 2013). Our findings indicate that social support from church members both offset and moderated (i.e. buffered) the associations between major discrimination experiences and both mental health outcomes. Moreover, these patterns were especially pronounced among those African Americans who received the highest levels of church-based support.

The experience of being denied equal treatment, accommodations, and resources in key institutional arenas – education, employment, housing, credit, and consumer services – can foster feelings of worthlessness, powerlessness, and hopelessness, which are important antecedents of depression. Such perceived discrimination can also generate worry and concern about the ability to achieve important life goals. How might congregational support processes ameliorate these consequences? Although data limitations prevent us from adjudicating among these various explanations, we see several plausible, albeit speculative, mechanisms or pathways. First, studies over the years have repeatedly highlighted the importance of reflected appraisals in shaping self-esteem. More recent work has argued that African American religious communities tend to encourage positive interactions among members and between members and clergy, and thus may bolster perceptions of self-worth and personal significance through positive reflected appraisals (Ellison 1993; Krause 2003). The perception that one is held in high regard by others whose assessments one values has been shown to elevate self-esteem, which may be especially important in the aftermath of unfavorable, disrespectful treatment by others.

Second, many, and perhaps most, African Americans still attend racially segregated congregations, and the Black Church is a repository of collective memory and group wisdom about racism and discrimination (Billingsley 1999; Lincoln and Mamiya 1990). Church-based support networks may permit individual victims of maltreatment to divulge their feelings freely, and to exchange ideas and experiences about how to respond to episodes of discrimination. This may facilitate bonding and solidarity with others who have undergone similar experiences, affirming that negative treatment stems from the ignorance of perpetrators, and does not reflect on the character or worth of victims. Such a process would be broadly consistent with recent literature linking religious factors with tendencies to cope by adopting benign reappraisals of stressful events and conditions (DeAngelis and Ellison 2017; Vishkin et al. 2016).

Third, although most discussions of church-based support processes have emphasized socioemotional assistance, church members may also benefit from instrumental aid (i.e., goods, services, and information) from fellow members and church programs (e.g., financial support, employment and housing assistance) (Billingsley 1999; Krause 2002, 2008a). This may be particularly helpful given that types of discrimination examined in this study tend to involve threats to financial security and the loss of other tangible resources and opportunities.

Fourth, in addition to focusing heavily on the mental and physical health benefits of socioemotional support, especially among African Americans, Krause (2002, 2008a) has developed the important construct of “spiritual support.” Although his work has centered primarily on older adults, one suspects that many of his core insights apply more broadly. In brief, spiritual support arises when church members help their fellows to apply religious ideals and scriptural insights to daily life, and to live out the tenets of their faith tradition. Moreover, Krause has also shown that social relations within the congregation – and particularly spiritual support – may encourage the cultivation of forgiveness (Krause and Ellison 2003) and other virtues or character strengths, including gratitude (Krause 2009) and meaning (Krause 2008b).

This is important in the present context because: (1) previous studies have shown that aspects of religion (including, but not restricted to, congregational social relations) are linked with tendencies toward forgiveness and other personal virtues (Krause and Ellison 2003); and (2) these virtues, in turn, are directly associated with improved mental health and can even moderate the effects of stressful life circumstances on psychosocial outcomes (Krause 2009; Toussaint, Worthington, and Williams 2015). These considerations hint at another potential explanation of why congregational support might buffer the psychosocial effects of discrimination, i.e., by encouraging forgiveness and forbearance, which may allow victims to release negative emotions such as anger and grief. Indeed, one recent study lends a degree of credence to this interpretation, revealing that African Americans who are more religious by certain indicators are more prone to respond to discrimination with forbearance (Hayward and Krause 2015). Support from church members may also give rise to feelings of gratitude, which have proven to elevate mood and divert focus away from unpleasant experiences (Krause 2009). Although we lack the data to test these and other promising explanations, they clearly warrant closer attention in future research.

We should also consider why both religious attendance and prayer failed to moderate the mental health effects of major discrimination in our models. For instance, previous studies (e.g. Bierman 2006; Ellison et al. 2008) found that religious attendance did buffer the adverse effects of discrimination on African Americans’ mental health (i.e. depression and psychological distress). There are at least two plausible explanations for these discrepancies. The first reason may involve variations in study design, i.e., differences in samples and populations, as well as in measures of discrimination and religious involvement. For one, both Bierman (2006) and Ellison and colleagues (2008) analyzed nationwide data whereas our study used data from Nashville. Bierman’s study also used only religious attendance and an omnibus measure of comfort-seeking from religion. One possibility is that Bierman’s finding regarding the buffering effects of religious attendance may actually have been gauging church-based support, in which case his measure of religious attendance was actually serving as a proxy for support. Likewise, while Bierman had a decent multi-item measure of discrimination, Ellison and colleagues had only a weak single-item measure of whether a respondent experienced a racist encounter within a month prior to the interview. In short, the differences in findings could reflect differences in measures of discrimination and religion, along with other variations in study design.

The focus on Nashville data, which stands in contrast to the more common practice of using nationwide probability samples, may also be important. Religious attendance levels may be above the national average in Nashville, and in the south more generally, in which case social conventions and cultural norms may shape religious attendance patterns to a greater extent than elsewhere (e.g. Ellison and Sherkat 1995; Hunt and Hunt 1999). Thus, for many southern African Americans, religious attendance may not be fundamentally about personal religious commitment. To the extent this is the case, it could help explain the null effects of attendance within our Nashville sample.

Why were there no main or buffering effects of frequency of prayer? The estimated net effects of prayer on mental health (both direct and stress-buffering) likely depend upon other factors, including (a) one’s images of and beliefs about God, (b) styles of attachment to God (e.g. secure, anxious, etc.), (c) modes of prayer (colloquial, petitionary, meditative), and (d) prayer expectancies, or beliefs about if and how God answers prayers (see Ellison et al. 2014). Unfortunately, our measure of prayer was too blunt of an instrument for gauging these distinctions. Future work would benefit from including these additional measures of God imagery, prayer modes and expectancies, and attachment styles to God.

Like most research, this study has several limitations, in addition to those noted above. First and most obviously, we have analyzed data from the African American subsample of the NSAHS, a cross-sectional survey of residents in Davidson County, TN. Because this is a community sample of rather modest size, the generalizability of these findings to other populations cannot be ascertained and awaits further research. In addition, the cross-sectional design of the NSAHS data makes it impossible to demonstrate causal relationships, in part because the temporal order among variables cannot be established. It is conceivable that alternative causal mechanisms are at work – e.g., more depressed and less satisfied individuals may be more prone to perceive or recall discriminatory events. Overall, however, we believe that the theoretical arguments developed here are much better suited to explain the empirical patterns reported in this study. Second, the measure of the key independent variable, church-based social support, consists only of a single item tapping the frequency with which congregation members provide assistance to the respondent. This is a rather blunt instrument, and therefore we cannot specify the levels of specific types of support that may have been received. Future research on the potential buffering role of church-based social support should distinguish between emotional, instrumental, and spiritual support to clarify the processes that are most effective at limiting the harmful psychosocial effects of discrimination.

Despite these limitations, our study adds additional evidence to discussions on the links between congregational social support and mental health among African Americans. Several studies have reported what appear to be salutary effects of church-based support on psychological well-being within this population, often among older adults (Chatters et al. 2011, 2015; Krause 2003, 2008a). Other recent work has called attention to the role of religious factors mitigating the emotional fallout from discrimination experiences (Bierman 2006; Ellison, Musick, and Henderson 2008), but to our knowledge only a few very recent empirical studies focused on church-based social support in this context (Head and Thompson 2017; Hope et al. 2017). Our study points to an apparently beneficial role of congregational support, particularly at high levels, in buffering the effects of major lifetime discrimination experiences on the mental health of African Americans. In addition to adjudicating the various explanations for these patterns that are discussed above, future studies might profitably examine the role of religious factors – church-based support as well as other dimensions, including specific beliefs – in dealing with perceived discrimination in informal encounters and everyday interactions, e.g., so-called “microaggressions.” Clearly discrimination against African Americans and other minority groups remains a grim reality in contemporary American society. In light of the findings reported here, and those of other recent work, continued efforts to clarify the complex role of religion in the process of coping with unfair and inequitable treatment should have a prominent place on the scholarly agenda.

## Figures and Tables

**Table 1.** Descriptive Statistics (*n*=627).

	Range	Mean (%)	SD	α
*Dependent Variables*				
Depression	1−4	1.69	0.48	.92
Life satisfaction	1−4	2.63	0.65	.79
*Focal Independent Variable*				
Major discrimination	0−7	1.18	1.42	
*Religious Involvement*				
Church attendance	0−3	2.01	1.02	
Prayer	1−6	5.01	1.32	
Religious social support	1−4	1.85	1.04	
Attends “weekly or more”	0−1	(44)		
Prays “several times a day”	0−1	(51)		
Receives support “very often”	0−1	(10)		
*Socio-demographics*				
Age	22−69	46.19	11.21	
Female	0−1	(53)		
Male (reference)	0−1	(47)		
Education (in years)	0−25	13.02	3.08	
Married	0−1	(30)		
Not married (reference)	0−1	(70)		
Employed	0−1	(66)		
Unemployed (reference)	0−1	(34)		
Household income	0−15	6.28	3.81	

**Table 2.** OLS Regression Models Estimating Past-Month Depressive Symptoms (*n*=627).

	(1)		(2)		(3)		(4)	
*Focal Variables*								
Major discrimination	0.061	**	0.061	*	0.062	*	0.060	*
Church attendance	0.019		0.019		0.018		0.019	
Prayer	−0.004		−0.004		−0.003		−0.004	
Religious social support	−0.028		−0.028		−0.028		−0.023	
*Interactions* [Major discrimination ✕…]								
Church attendance			−0.002					
Prayer					−0.003			
Religious social support							−0.025	*
*Socio-demographics*								
Age	−0.008	***	−0.008	***	−0.008	***	−0.008	***
Female	0.135	*	0.134	*	0.134	*	0.131	*
Education	−0.006	*	−0.006	*	−0.006	*	−0.006	*
Married	−0.040		−0.039		−0.040		−0.041	
Employed	−0.153	**	−0.153	**	−0.152	**	−0.157	**
Household income	−0.025	**	−0.025	**	−0.025	**	−0.024	**

Constant	2.294		2.388		2.337		2.303	
R^2^	0.184		0.184		0.184		0.189	

*Note*: models adjust for cluster sampling by block group.

* p < .05

** p < .01

*** p < .001 (two-tailed test).

**Table 3.** OLS Regression Models Estimating Life Satisfaction (*n*=627).

	(1)		(2)		(3)		(4)	
*Focal Variables*								
Major discrimination	−0.061	✝	−0.060		−0.066	*	−0.060	✝
Church attendance	0.013		0.014		0.014		0.012	
Prayer	0.035		0.034		0.033		0.035	
Religious social support	0.040	*	0.040	*	0.040	✝	0.035	✝
*Interactions* [Major discrimination ✕…]								
Church attendance			−0.009					
Prayer					0.010			
Religious social support							0.025	
*Socio-demographics*								
Age	0.002		0.002		0.002		0.002	
Female	−0.012		−0.013		−0.010		−0.009	
Education	−0.002		−0.001		−0.001		−0.001	
Married	0.119		0.121		0.119		0.121	
Employed	−0.043		−0.043		−0.045		−0.038	
Household income	0.045	**	0.044	**	0.045	**	0.044	**

Constant	2.087		2.051		2.187		2.101	
R^2^	0.120		0.121		0.121		0.124	

*Note*: models adjust for cluster sampling by block group.

✝ p < .10

* p < .05

** p < .01

*** p < .001 (two-tailed test).

**Table 4.** OLS Regression Models Estimating Religious Stress-Moderating Thresholds (*n*=627).

	Depression						Life Satisfaction					
		
	(1)		(2)		(3)		(4)		(5)		(6)	
*Direct Associations*												
Major discrimination	0.067	*	0.048		0.069	**	−0.069		−0.049		−0.071	*
Attends “weekly or more”	−0.028		−0.029		−0.029		0.084		0.087		0.087	
Prays “several times a day”	0.023		0.017		0.020		0.038		0.043		0.042	
Receives support “very often”	−0.137	*	−0.135	*	−0.118	*	0.099	***	0.098	***	0.074	*
*Interactions* [Major discrimination ✕…]												
Attends “weekly or more”	−0.015						0.023					
Prays “several times a day”			0.027						−0.022			
Receives support “very often”					−0.071	*					0.093	*

Constant	2.303		2.308		2.301		2.214		2.208		2.216	
R^2^	0.190		0.191		0.194		0.118		0.118		0.122	

*Note*: models adjust for cluster sampling by block group and socio-demographics.

* p < .05

** p < .01

*** p < .001 (two-tailed test).

## References

[R1] Aiken, Leona S, West Stephen G, and Reno Raymond R. 1991. Multiple Regression: Testing and Interpreting Interactions. California: Sage.

[R2] Berger Maximus, and Saranyai Zoltan. 2015. More than Skin Deep: Stress Neurobiology and Mental Health Consequences of Racial Discrimination. Stress 18: 1–10. 25407297 10.3109/10253890.2014.989204

[R3] Berger Peter L. 1967. The Sacred Canopy. Garden City, NY: Doubleday.

[R4] Bertrand Marianne, and Mullainathan Sendhil. 2004. Are Emily and Greg More Employable than Lakisha and Jamal? A Field Experiment on Labor Market Discrimination. American Economic Review 94: 991–1013.

[R5] Bierman Alex. 2006. Does Religion Buffer the Effects of Discrimination on Mental Health? Differing Effects by Race. Journal for the Scientific Study of Religion 45: 551–565.

[R6] Billingsley Andrew. 1999. Mighty Like a River: The Black Church and Social Reform. New York: Oxford University Press.

[R7] Chatters Linda M., Bullard Kai M., Taylor Robert J., Woodward Amanda T., Neighbors Harold W., and Jackson James S.. 2008. Religious Participation and DSM-IV Disorders among Older African Americans: Findings from the National Survey of American Life (NSAL). American Journal of Geriatric Psychiatry 16: 957–965. 10.1097/JGP.0b013e3181898081 PMC2631206 19038894

[R8] Chatters Linda M., Taylor Robert J., Bullard Kai M., and Jackson James S.. 2009. Race and Ethnic Differences in Religious Participation: African Americans, Caribbean Blacks, and non-Hispanic Whites. Ethnic and Racial Studies 32: 1143–1163. 20975850 10.1080/01419870802334531 PMC2962581

[R9] Chatters Linda M., Taylor Robert J., Lincoln Karen D., Nguyen Ann, and Joe Sean. 2015. Church- based Social Support and Suicidality among African Americans and Black Caribbeans. Archives of Suicide Research 15: 337–353. 10.1080/13811118.2011.615703 22023642

[R10] Chatters Linda M., Taylor Robert J., Woodward Amanda T., and Nicklett Emily J.. 2011. Social Support from Church Members and Family Members and Depressive Symptoms among Older African Americans. American Journal of Geriatric Psychiatry 23: 559–567. 10.1016/j.jagp.2014.04.008 PMC4216772 24862679

[R11] Cooper-Lewter Nicholas C., and Mitchell Henry H.. 1986. Soul Theology: The Heart of American Black Culture. San Francisco: Harper and Row.

[R12] DeAngelis Reed T., and Ellison Christopher G.. 2017. Kept in His Care: The Role of Perceived Divine Control in Positive Reappraisal Coping. Religions 8(8): 133.

[R13] Diener Ed, Emmons Robert A., Larsen Randy J., and Green Sharon. 1985. The Satisfaction with Life Scale. Journal of Personality Assessment 49(1): 71–75. 16367493 10.1207/s15327752jpa4901_13

[R14] Edwards Korie. 2009. Race, Religion, and Worship: Are African American Worship Practices Distinct? Journal for the Scientific Study of Religion 48: 30–52.

[R15] Ellison Christopher G. 1991. Religious Involvement and Subjective Well-being. Journal of Health and Social Behavior 32: 80–99. 2007763

[R16] Ellison Christopher G. 1993. Religious Involvement and Self-Perceptions among Black Americans. Social Forces 71: 1027–1055.

[R17] Ellison Christopher G., Bradshaw Matt, Flannelly Kevin J., and Galek Kathleen C.. 2014. Prayer, Attachment to God, and Symptoms of Anxiety-related Disorders among U.S. Adults. Sociology of Religion 75: 208–233.

[R18] Ellison Christopher G., and Flannelly Kevin J.. 2009. Religious Involvement and Risk of Major Depression in a Prospective Nationwide Study of African American Adults. Journal of Nervous and Mental Disease 197(8): 568–573. 19684492 10.1097/NMD.0b013e3181b08f45

[R19] Ellison Christopher G., and Gay David A.. 1990. Region, Religious Involvement, and Life Satisfaction among Black Americans. The Sociological Quarterly 31: 123–147.

[R20] Ellison Christopher G., and George Linda K.. 1994. Religious Involvement, Social Ties, and Social Support in a Southeastern Community. Journal for the Scientific Study of Religion 33: 46–61.

[R21] Ellison Christopher G., and Henderson Andrea K. 2011. Religion and Mental Health: Through the Lens of the Stress Process. Pp. 11–44 in Toward a Sociological Theory of Religion and Health, edited by Blasi Anthony J.. Boston: Brill.

[R22] Ellison Christopher G., Hummer Robert A., Burdette Amy M., and Benjamins Maureen R.. 2010. Race, Religious Involvement, and Health: The Case of African Americans. Pp. 321–348 in Religion, Families, and Health: Population-based Research in the United States, edited by Ellison Christopher G. and Hummer Robert A.. New Brunswick, NJ: Rutgers University Press.

[R23] Ellison Christopher G., Musick Marc A., and Henderson Andrea K.. 2008. Balm in Gilead: Racism, Religious Involvement, and Psychological Distress among African American Adults. Journal for the Scientific Study of Religion 47: 291–309.

[R24] Ellison Christopher G., and Sherkat Darren E.. 1995. The Semi-Involuntary Institution Revisited: Regional Variations in Church Participation among Black Americans. Social Forces 73: 1415–1437.

[R25] Ellison Christopher G., and Taylor Robert J.. 1996. Turning to Prayer: Social and Situational Antecedents of Religious Coping among African Americans. Review of Religious Research 38: 111–131.

[R26] Devin English, Lambert Sharon F., and Ialongo Nicholas S.. 2014. Longitudinal Associations between Experienced Racial Discrimination and Depressive Symptoms in African American Adolescents. Developmental Psychology 50: 1190–1196. 24188037 10.1037/a0034703 PMC4501852

[R27] Fix Michael, and Turner Margery Austin. 1999. National Report Card on Discrimination in America. Washington: Urban Institute.

[R28] Gilkes Cheryl Townsend. 1980. The Black Church as a Therapeutic Community: Suggested Areas for Research into the Black Religious Experience. Journal of the Interdenominational Theological Center 8: 29–44.

[R29] Griffith, Ezra EH, Thelma English, and Viola Mayfield. 1980. Possession, Prayer, and Testimony: Therapeutic Elements of the Wednesday Night Prayer Meeting in a Black Church. Psychiatry 43: 120–128. 7384305 10.1080/00332747.1980.11024057

[R30] Harris Anne-Marie G., Harrison Gerald R., and Williams Jerome D.. 2005. Courting Customers: Assessing Consumer Racial Profiling and Other Marketplace Discrimination. Journal of Public Policy and Marketing 24: 163–171.

[R31] David Hayward R., and Krause Neal. 2015. Religion and Strategies for Coping with Racial Discrimination among African Americans and Caribbean Blacks. International Journal of Stress Management 22: 70–91.

[R32] Head Rachel N., and Maxine Seaborn Thompson. 2017. Discrimination-related Anger, Religion, and Distress: Differences between African Americans and Caribbean Black Americans. Society and Mental Health.

[R33] Henderson Andrea K. 2016. The Long Arm of Religion: Childhood Adversity, Religion, and Self- Perception among Black Americans. Journal for the Scientific Study of Religion 55: 324–348.

[R34] Himmelstein Mary S., Young Danielle M., Sanchez Diana T., and Jackson James S.. 2015. Vigilance in the Discrimination-Stress Model for Black Americans. Psychology and Health 30: 253–267. 25247925 10.1080/08870446.2014.966104 PMC4434586

[R35] Holt Cheryl L., Schulz Emily, Williams Beverly R., Clark Eddie M., and Wang Min Qi. 2014. Social Support as a Mediator of Religious Involvement and Physical and Emotional Functioning in a National Sample of African Americans. Mental Health, Religion, and Culture 17: 421–435.

[R36] Hope Meredith O., Assari Shervin, Yasmin C. Cole-Lewis, and Caldwell Cleopatra H.. 2017. Religious Social Support, Discrimination, and Psychiatric Disorders among Black Adolescents. Race and Social Problems 9: 102–114. 32089748 10.1007/s12552-016-9192-7 PMC7034935

[R37] Hunt Larry L., and Hunt Matthew O.. 1999. Regional Patterns of African American Church Attendance: Revisiting the Semi-Involuntary Thesis. Social Forces 78(2): 779–791.

[R38] Idler Ellen L., Musick Marc A., Ellison Christopher G., George Linda K., Krause Neal, Ory Marcia, Pargament Kenneth, Powell Lynda, Underwood Lynn G., and Williams David R.. 2003. Measuring Multiple Dimensions of Religion and Spirituality for Health Research: Conceptual Background and Findings from the 1998 General Social Survey. Research on Aging 25: 327–365.

[R39] Jang Sung-Joon, and Byron Johnson R.. 2004. Exploring Religious Effects on Distress among African Americans. Journal for the Scientific Study of Religion 43: 239–260.

[R40] Kessler Ronald C., Mickelson Kristin D., and Williams David R.. 1999. The Prevalence, Distribution, and Mental Health Consequences of Perceived Discrimination in the United States. Journal of Health and Social Behavior 40: 208–230. 10513145

[R41] Koenig Harold G., Carson Velma, and King Dana. 2012. Handbook of Religion and Health, 2nd ed. New York: Oxford University Press.

[R42] Krause Neal. 2002. Exploring Race Differences in a Comprehensive Battery of Church-based Social Support Measures. Review of Religious Research 44: 126–149.

[R43] Krause Neal. 2003. Exploring Race Differences in the Relationships between Social Interaction with Clergy and Feelings of Self-Worth in Late Life. Sociology of Religion 64: 183–205.

[R44] Krause Neal. 2004a. Common Facets of Religion, Unique Facets of Religion, and Life Satisfaction among Older African Americans. Journal of Gerontology: Social Sciences 59B: S109–S117. 10.1093/geronb/59.2.s109 15014098

[R45] Krause Neal. 2004b. Assessing the Relationships among Prayer Expectations, Race, and Self-Esteem in Late Life. Journal for the Scientific Study of Religion 43: 395–408.

[R46] Krause Neal. 2006. Exploring the Effects of Church-based and Secular Social Support on Self-Rated Health in Late Life. Journal of Gerontology: Social Sciences 61B: S35–S43. 10.1093/geronb/61.1.s35 16399948

[R47] Krause Neal. 2008a. Aging in the Church: How Social Relationships Affect Health. West Conshohocken, PA: Templeton Press.

[R48] Krause Neal. 2008b. The Social Foundation of Religious Meaning in Life. Research on Aging 30: 395–427.

[R49] Krause Neal. 2009. Religious Involvement, Gratitude, and Changes in Depressive Symptoms over Time. International Journal for the Psychology of Religion 19: 155–172. 20333271 10.1080/10508610902880204 PMC2843928

[R50] Krause Neal, and Chatters Linda M.. 2005. Exploring Race Differences in a Multidimensional Battery of Prayer Measures among Older Adults. Sociology of Religion 66: 23–43.

[R51] Krause Neal, and Ellison Christopher G.. 2003. Forgiveness by God, Forgiveness of Others, and Psychological Well-being in Late Life. Journal for the Scientific Study of Religion 42: 77–93. 21373377 10.1111/1468-5906.00162 PMC3046863

[R52] Kwate Naa Oyo, and Goodman Melody S.. 2015. Cross-sectional and Longitudinal Effects of Racism on Mental Health among Residents of Black Neighborhoods in New York City. American Journal of Public Health 105: 711–718. 25521873 10.2105/AJPH.2014.302243 PMC4358177

[R53] Levin Jeffrey S., Chatters Linda M., and Taylor Robert J.. 1995. Religious Effects on Health and Life Satisfaction among Black Americans. Journal of Gerontology: Social Sciences 50: S154–S163. 10.1093/geronb/50b.3.s154 7767699

[R54] Lim Chaeyoon, and Putnam Robert D.. 2010. Religion, Social Networks, and Life Satisfaction. American Sociological Review 75: 914–933.

[R55] Lincoln C. Eric, and Lawrence H. Mamiya. 1990. The Black Church in the African American Experience. Durham, NC: Duke University Press.

[R56] Mattis Jacqueline S., and Jagers Robert. 2001. A Relational Framework for the Study of Religiosity and Spirituality in the Lives of African Americans. Journal of Community Psychology 29: 519–539.

[R57] Neighbors Harold W., Musick Marc A., and Williams David R.. 1998. The African American Minister as a Source of Help for Serious Personal Crises: Bridge or Barrier to Mental Health Care? Health Education and Behavior 25: 759–777. 9813746 10.1177/109019819802500606

[R58] Nguyen Ann, Chatters Linda M., and Taylor Robert J.. 2016. African American Extended Family and Church-based Support Network Typologies. Family Relations 65: 701–715. 28479650 10.1111/fare.12218 PMC5417543

[R59] Oates Gary L., and Goode Jennifer. 2013. Racial Differences in the Effects of Religiosity and Mastery on Psychological Distress: Evidence from National Longitudinal Data. Society and Mental Health 3: 40–58. 23762783 10.1177/2156869312455930 PMC3677831

[R60] Pager Devah, and Shepherd Hana. 2008. The Sociology of Discrimination: Racial Discrimination in Employment, Housing, Credit, and Consumer Markets. Annual Review of Sociology 34: 181–209. 10.1146/annurev.soc.33.040406.131740 PMC2915460 20689680

[R61] Pargament Kenneth I. 1997. The Psychology of Religion and Coping. New York: Guilford.

[R62] Pargament Kenneth I., Koenig Harold G., and Perez Lisa. 2000. The Many Methods of Religious Coping: Development and Initial Validation of the RCOPE. Journal of Clinical Psychology 56: 519–543. 10775045 10.1002/(sici)1097-4679(200004)56:4<519::aid-jclp6>3.0.co;2-1

[R63] Pollner Melvin L. 1989. Divine Relations, Social Relations, and Well-being. Journal of Health and Social Behavior 30: 92–104. 2470806

[R64] Radloff Lenore S. 1977. The CES-D Scale: A Self-Report Depression Scale for Research in the General Population. Applied Psychological Measurement 1(3): 385–401.

[R65] Roberts J Deotis. 2005. Liberation and Reconciliation: A Black Theology, 2nd ed. Louisville: Westminster John Knox.

[R66] Roscigno Vincent J. 2007. The Face of Discrimination: How Race and Gender Impact Work and Home Lives. Lanham, MD: Rowman and Littlefield.

[R67] Ross Stephen L., and Yinger John. 2002. The Color of Credit: Mortgage Discrimination, Research Methodology and Fair Lending Enforcement. Cambridge, MA: MIT Press.

[R68] Schieman Scott, Bierman Alex, and Ellison Christopher G.. 2013. Religion and Mental Health. Pp. 457–478 in Handbook of the Sociology of Mental Health, edited by Aneshensel Carol S., Phelan Jo C., and Bierman Alex. Netherlands: Springer.

[R69] Sharp Shane. 2010. How Does Prayer Help Manage Emotions? Social Psychology Quarterly 73: 417–437.

[R70] Tabak Melanie A., and Mickelson Kristin D.. 2009. Religious Service Attendance and Distress: The Moderating Role of Stressful Life Events and Race/Ethnicity. Sociology of Religion 70: 149–164.

[R71] Taylor Robert J., Chatters Linda M., and Abelson Jamie M.. 2012. Religious Involvement and DSM- IV 12-Month and Lifetime Major Depressive Disorder among African Americans. Journal of Nervous and Mental Disease 200: 856–862. 22986280 10.1097/NMD.0b013e31826b6d65 PMC3464345

[R72] Taylor Robert J., Chatters Linda M., and Joe Sean. 2011. Religious Involvement and Suicidal Behavior among African Americans and Black Caribbeans. Journal of Nervous and Mental Disease 199: 478–486. 21716062 10.1097/NMD.0b013e31822142c7 PMC3128792

[R73] Taylor Robert J., Chatters Linda M., and Levin Jeffrey S.. 2004. Religion in the Lives of African Americans: Social, Psychological, and Health Perspectives. Thousand Oaks, CA: Sage.

[R74] Taylor Robert J., Ellison Christopher G., Chatters Linda M., Levin Jeffrey S., and Lincoln Karen D.. 2000. Mental Health Services within Faith Communities: The Role of Clergy in Black Churches. Social Work 45: 73–87. 10634088 10.1093/sw/45.1.73

[R75] Taylor Robert J., Thornton Michael C., and Chatters Linda M.. 1987. Blacks’ Perceptions of the Sociohistorical Role of the Church. Journal of Black Studies 18: 123–138.

[R76] Toussaint Loren, Worthington Everett, and Williams David R. (eds.). 2015. Forgiveness and Health: Scientific Evidence and Theories Relating Forgiveness to Better Health. New York: Springer.

[R77] Tsitsos William. 2003. Race Differences in Congregational Social Service Activity. Journal for the Scientific Study of Religion 42: 205–215.

[R78] Vishkin Allon, Bigman Yochana E., Porat Roni, Solak Nevin, Halperin Eran, and Tamir Maya. 2016. God Rest our Hearts: Religiosity and Cognitive Reappraisal. Emotion 16(2): 252–262. 26461249 10.1037/emo0000108

[R79] Washington Joseph M. 1994. Conversations with God: Two Centuries of Prayers by African Americans. New York: Harper.

[R80] White Ian R., Royston Patrick, and Wood Angela M.. 2011. “Multiple Imputation Using Chained Equations: Issues and Guidance for Practice.” Statistics in Medicine 30(4): 377–99. 21225900 10.1002/sim.4067

[R81] Williams David R., and Mohammed Selina A.. 2009. Discrimination and Racial Disparities in Health: Evidence and Needed Research. Journal of Behavioral Medicine 32: 20–47. 19030981 10.1007/s10865-008-9185-0 PMC2821669

[R82] Williams David R., and Williams-Morris R. 2000. Racism and Mental Health: The African American Experience. Ethnicity and Health 5(3/4): 243–268. 11105267 10.1080/713667453

[R83] Williams Richard A., Nesiba Reynold, and Eileen Diaz McConnell. 2005. The Changing Face of Inequality in Home Mortgage Lending. Social Problems 52: 181–208.

